# Force–frequency relationship during fatiguing contractions of rat medial gastrocnemius muscle

**DOI:** 10.1038/s41598-020-68392-6

**Published:** 2020-07-14

**Authors:** Keenan B. MacDougall, Andrea N. Devrome, Anders M. Kristensen, Brian R. MacIntosh

**Affiliations:** 1 Faculty of Kinesiology, University of Calgary, Calgary, AB T2N 1N4 Canada; 2 Faculty of Medicine, University of Calgary, Calgary, AB T2N 1N4 Canada; 3 Department of Public Health, Aarhus University, Aarhus C, Denmark

**Keywords:** Muscle contraction, Cell biology

## Abstract

The force–frequency relationship presents the amount of force a muscle can produce as a function of the frequency of activation. During repetitive muscular contractions, fatigue and potentiation may both impact the resultant contractile response. However, both the apparent fatigue observed, and the potential for activity-dependent potentiation can be affected by the frequency of activation. Thus, we wanted to explore the effects that repetitive stimulation had on the force–frequency relationship. The force–frequency relationship of the rat medial gastrocnemius muscle was investigated during consecutive bouts of increasing fatigue with 20 to 100 Hz stimulation. Force was measured prior to the fatiguing protocol, during each of three levels of fatigue, and after 30 min of recovery. Force at each frequency was quantified relative to the pre-fatigued 100 Hz contractions, as well as the percentage reduction of force from the pre-fatigued level at a given frequency. We observed less reduction in force at low frequencies compared to high frequencies, suggesting an interplay of fatigue and potentiation, in which potentiation can “protect” against fatigue in a frequency-dependent manner. The exact mechanism of fatigue is unknown, however the substantial reduction of force at high frequency suggests a role for reduced force per cross-bridge.

## Introduction

The magnitude of force a muscle can produce at any given time is dependent on a variety of factors, including: sarcomere length, velocity of shortening, frequency of stimulation, as well as the history of activation. Many of these factors may be acting simultaneously, leading to a complex interplay in which the muscular force output is dependent upon multiple mechanisms, some of which are cooperative, and some of which counteract one another. This paper will look at the interaction of the last two factors listed above: the frequency of stimulation, and the history of activation.

The force–frequency relationship refers to the phenomenon in which the isometric force produced by a muscle increases with the frequency of activation, resulting in a sigmoidal curve which plateaus at the high frequencies. At higher frequencies of activation, more Ca^2+^ is released from the sarcoplasmic reticulum resulting in increased muscular force, until a “saturation” effect is seen in which after a certain point, more Ca^2+^ release will not result in increased force^1,2^. Naturally, the force–frequency relationship will look very similar to the force–pCa relationship, as [Ca^2+^] is essentially the deciding factor in both of these relationships.

The history of activation can also affect subsequent force generation in two general ways; after previous contractile activity, *decreased* force output will be the result of muscular fatigue, whereas *increased* force output will be the result of activity dependent potentiation. Fatigue can be broadly defined as “a response that is less than the expected or anticipated contractile response, for a given stimulation”^3,4^. Activity dependent potentiation, on the other hand, is essentially the opposite: “an enhanced contractile response which results from prior contractile activity”^3^. Whether fatigue or potentiation plays a larger role in the subsequent force output is dependent on the specifics of the prior contractile activity and the conditions of the current contraction.

The mechanisms of muscle fatigue are dependent upon multiple factors related to the exercise or stimulation protocol. Impairments at any level of the excitation–contraction coupling have the potential to decrease the resultant muscle force; for example, reductions in motor neuron firing rate^5,6^, failure at the neuromuscular junction^7^, and accumulation of metabolites such as H^+^ and inorganic phosphate (P_i_)^8,9^ have all been implicated in muscle fatigue, and indeed different mechanisms have the potential to coexist. Nonetheless, the specific “downstream” effects of these factors can be broadly categorized into two groups: (1) reduced number of engaged cross-bridges or (2) less force per cross-bridge. Lower average myoplasmic [Ca^2+^], or decreased Ca^2+^ sensitivity can cause fewer active cross-bridges^10^, while alterations to the molecular interaction of the cross-bridge with actin can reduce the force per individual cross-bridge^11^. Overall, lower average myoplasmic [Ca^2+^] seems to be the primary factor involved in most forms of fatigue^3,12,13^.

Just as fatigue might negatively affect the force output via modulating Ca^2+^ release, Ca^2+^ sensitivity, or cross bridge function, activity-dependent potentiation might *enhance* force output by regulating these same factors; namely, increased Ca^2+^ release, increased Ca^2+^ sensitivity, or enhanced cross-bridge function. The mechanism for activity-dependent potentiation is most often considered to be due to the phosphorylation of the myosin regulatory light chains (RLC) by myosin light chain kinase (MLCK)^14–17^. Enhanced Ca^2+^ release leading to increased peak [Ca^2+^]^13,18^, as well as potentiation without RLC phosphorylation^19^ have also been observed. RLC phosphorylation is thought to increase the mobility of the myosin head, allowing it to move away from the myosin backbone, closer to the actin filament^20^. This enhanced mobility and proximity of contractile proteins increases the probability of cross-bridge formation, increasing the rate of attachment and thereby increasing force output at less than maximal Ca^2+^ concentration. Therefore, the enhanced force production from activity-dependent potentiation seems to be due primarily to increased Ca^2+^ sensitivity^3^.

Ca^2+^ on its own can also effectively increase myosin head mobility by binding to the myosin molecule itself^21^. At submaximal [Ca^2+^], there is little binding of Ca^2+^ to myosin, so increased mobility is not apparent. At maximal activation, Ca^2+^ can bind to myosin, moving the myosin head away from the backbone, which produces an effect similar to RLC phosphorylation by increasing the probability of cross-bridge interaction with actin. Seen this way, it is easy to understand why potentiation associated with RLC phosphorylation is not apparent during maximal activation. The myosin head is already situated away from the myosin filament backbone, and so RLC phosphorylation becomes redundant^22^.

The frequency of activation is an important determinant of the force produced by a muscle. Fatigue and potentiation can modulate the force differently at different frequencies. The history of activation will affect not only the level of fatigue, but also the level of potentiation. It is a fascinating scenario to determine how these factors interact. Indeed, fatigue and potentiation have been shown to co-exist, creating difficulty in predicting the contractile response during intermittent contractions^3,23,24^. The aim of this study was to determine how the force–frequency relationship was affected *during* consecutive bouts of increasingly fatiguing contractions. It was hypothesized that force production would decrease as fatigue increased, but due to the frequency dependence of potentiation, the relative reduction in force during fatigue would be dependent on the stimulation frequency.

## Methods

### Muscle preparation

Female Sprague Dawley rats (~ 250–400 g) were fed standard rat chow and water ad libitum. All procedures followed in this study were approved by the University of Calgary Animal Care Committee. Consistent with the guidelines of the Canadian Council on Animal Care, only approved procedures were followed in conducting these experiments. The rats (n = 7) were anaesthetized with 2% isoflurane in oxygen and had their left hindlimb surgically prepared for in situ measurement of force of the medial gastrocnemius muscle^25^. An incision was made from the calcaneus to the spine, and the subcutaneous connective tissue was cleared away. The superficial muscles were cut to expose the sciatic nerve. With the aid of a dissecting microscope and microstimulation, nerves innervating any muscles other than the medial gastrocnemius were cut, to ensure that the medial gastrocnemius was the only muscle activated. A string was tied around the sciatic nerve proximally, and the nerve was cut above this. The triceps surae muscle was further dissected, and the tendons of the plantaris, soleus, and lateral gastrocnemius were cut from the calcaneus, again to ensure that all measured force would be from the medial gastrocnemius. A silk string was tied around the Achilles tendon, and then the calcaneus was cut from the rest of the foot, leaving a small piece of the calcaneus attached to the Achilles tendon to prevent the string from slipping off. The foot and lower leg were then amputated by cutting through the tibia about midway up the bone. A metal probe was placed in the open end of the tibia, and another was inserted into the femur perpendicularly after drilling a small pilot hole. The string attached to the Achilles tendon was attached to a force transducer via a stainless steel wire, and the metal probes inserted into the tibia and femur were secured onto a metal frame to immobilize the rat’s leg during the experiment. The metal frame was attached to a stepper motor (Model MD2, Arrick Robotics Systems, Hurst, TX), that allowed the muscle–tendon length to be changed. The rat’s skin around its lower limb was pulled up and attached to the metal frame by elastic bands to create a bath filled with warm mineral oil, and the muscle was kept at physiological temperature with an infrared heat lamp and tin foil shields. Rectal and oil bath temperatures were kept between 36 and 38 °C. The cut sciatic nerve still attached to the medial gastrocnemius was inserted into an electrode cuff connected to a stimulator (Grass Instruments). All stimulation protocols used supramaximal square wave pulses with a 50 μs pulse width, similar to previous work^19,25^.

### Stimulation protocol

**Figure 1:**
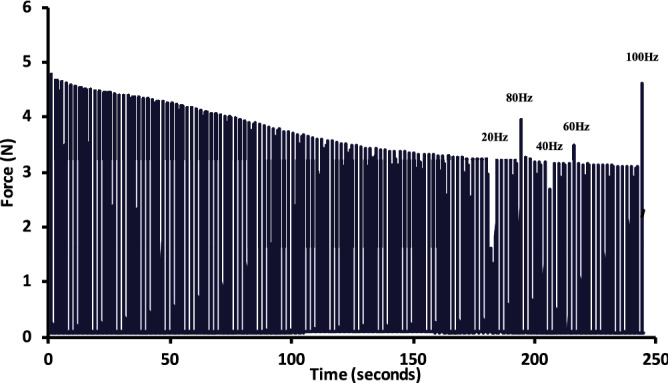
Example force tracing showing force reduction during a fatiguing protocol consisting of 50 Hz contractions every 2 s. Once force stabilized, test contractions were done in a randomized order to obtain a force–frequency relationship during fatigue. The test contractions are shown with the stimulation frequency above.

Stimulation voltage was established by stimulating the nerve with single pulses while decreasing the stimulation voltage. The lowest voltage that resulted in 100% of twitch force was then doubled for use during the experiment. This supramaximal voltage ensures that all responsive motor units were being recruited. Optimum length was then found by stimulating with double pulses at 200 Hz while varying the muscle–tendon length in 1 mm increments. The active force (total force minus resting force) was calculated for each length, and the length that corresponded to the highest active force was used for the rest of the experiment. Once the optimum length was found, an initial force–frequency protocol was completed in an unfatigued state by stimulating the nerve with 50 µs monophasic square pulses at 20 Hz, 40 Hz, 60 Hz, 80 Hz, and 100 Hz for 200 ms with 5 min recovery between contractions to minimize fatigue and potentiation. The order of presentation of these frequencies was randomized prior to each experiment. After this initial protocol, 3 fatiguing stimulation sequences were done consecutively (with no recovery time between sequences), consisting of 200 ms 50 Hz contractions, until the contractile force reached a steady-state. The first sequence consisted of 1 contraction every 4 s. The second sequence consisted of 1 contraction every 2 s, and the third consisted of 1 contraction every second. Once the fatigue reached a steady state, contractions were obtained at frequencies between 20 and 100 Hz in the same random order that had been determined at the beginning of that experiment. Steady-state was determined by a visual inspection of the force–time series, and taken as a point in which the change in force over the previous 60 s was less than 5%. Twitch contractions are considered 1 Hz and were measured from the first rise and fall of force during the 20 Hz contractions. The test contractions intermittently replaced the ongoing 50 Hz contractions (see Fig. 1), to obtain a force–frequency relationship at each level of increasing fatigue. After the final fatigue protocol was finished, a 30-min rest period was given to let the muscle recover. After recovery, a final force–frequency stimulation protocol was completed in the same order as in the pre-fatigued state, with 5 min recovery between contractions. This final sequence of measurements allowed us to determine if there was any evidence of long-lasting fatigue.

### Data collection and analysis

All data were collected via a PowerLab data acquisition device and LabChart software (ADI Instruments, Colorado Springs, USA) at a sampling rate of 2000 Hz. Data were then exported to Microsoft Excel (Microsoft Corporation, Redmond, USA) and IBM SPSS (IBM, Armonk, USA), for further analysis. Active force for each contraction was calculated by subtracting the initial passive force from the peak force. Force at each fatigue level was then expressed in two ways: normalized relative to the force produced in the pre-fatigued 100 Hz contraction, as well as the percentage reduction in normalized force relative to the pre-fatigued contraction at a given stimulation frequency. The latter was calculated by taking the active force relative to the pre-fatigued contraction at a given frequency, and then subtracting that value from 100%. A repeated measures two-way-ANOVA was done for each measure to determine differences in relative active force and the percentage reduction in active force across stimulation frequencies and fatigue levels. As we were primarily interested in the frequency-dependence of fatigue, and not on the frequency dependence of force, if a significant interaction was seen, one-way repeated measures ANOVA tests were performed to determine if there were significant differences in force between levels of fatigue at a given stimulation frequency. Pairwise comparisons of simple main effects were corrected with a Bonferroni adjustment. Significance level was set at 0.05.

## Results

**Figure 2:**
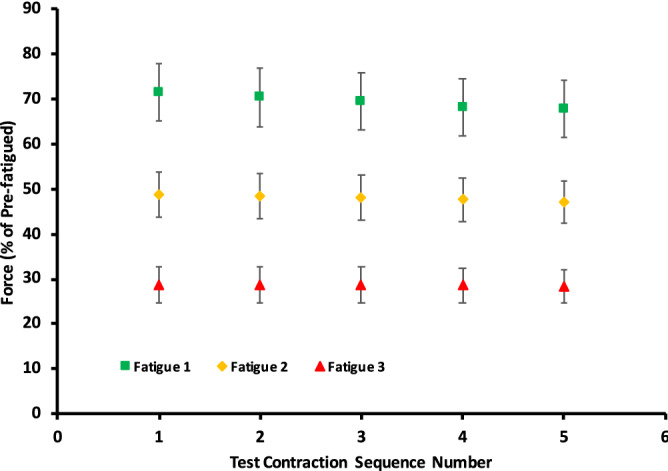
Mean relative force output of the 50 Hz contractions, immediately preceding each test contraction, and expressed relative to the pre-fatigued 50 Hz contraction. This graph shows the constancy of force over each measurement period. Fatigue 1 = 1 contraction every 4 s. Fatigue 2 = 1 contraction every 2 s. Fatigue 3 = 1 contraction every second. *Each subsequent protocol resulted in a significant reduction in force compared to the previous protocol. Data points are mean ± SEM.

Figure 1 shows a force–time series of an example fatiguing protocol, from the onset of the 50 Hz contractions until the end of the series of test contractions. From the beginning of the protocol until a steady-state was reached and the test contractions began, 50 Hz active force further decreased to 70 ± 16, 48 ± 12, and 29 ± 10% of the initial active force during the first, second and third fatiguing protocols, respectively (mean ± SD) (see Fig. 2). The average time in minutes to reach steady state after beginning each successive protocol was 7:17 ± 2:53, 3:52 ± 1:05, and 3:01 ± 1:05 for the first, second and third fatiguing protocols, respectively (mean ± SD).

**Figure 3:**
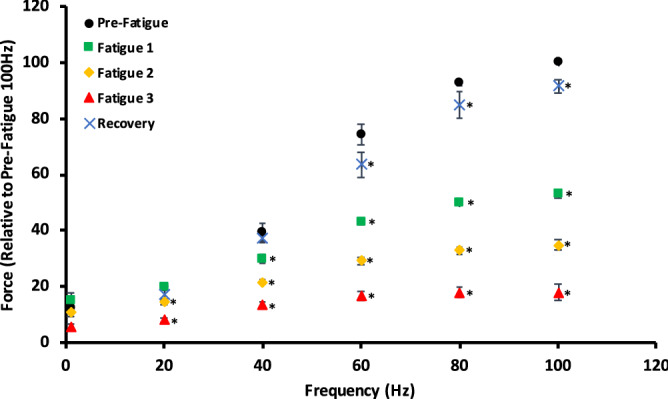
Active force expressed relative to the pre-fatigued 100 Hz contraction for control, three levels of fatigue, and after 30 min of recovery. Symbols/bars are mean ± SE. When no error bar is visible, it is within the symbol. Twitch contractions are shown near 0 frequency. Fatigue 1 = 1 contraction every 4 s. Fatigue 2 = 1 contraction every 2 s. Fatigue 3 = 1 contraction every second. *Denotes a significant difference from the pre-fatigue value at that frequency.

Two-way repeated measures ANOVA revealed significant interaction between frequency of stimulation and level of fatigue on the relative force output (*p* < 0.001). Simple main effects of fatigue level were then determined by repeated measures one-way ANOVA tests, and revealed a frequency-dependence of the degree of force loss over the successive fatigue protocols. Figure 3 shows the active force relative to the maximal 100 Hz force for each frequency and fatigue condition. Twitch active force was not significantly different from pre-fatigue levels at all subsequent levels of fatigue, or post-recovery. At the first level of fatigue, 20 Hz active force was not significantly different from pre-fatigue values, but significant reductions were seen at the second and third levels of fatigue. For 60 Hz, 80 Hz, and 100 Hz contractions, there were significant differences in force from the pre-fatigue values at all subsequent levels of fatigue, and after 30 min of recovery. Evidently, at lower stimulation frequencies, it took a larger degree of fatigue for losses of force to be apparent (e.g. the twitch active force never significantly decreased below the pre-fatigue values, and the 20 Hz active force was not significantly decreased from pre-fatigue values until the second fatiguing protocol). In contrast, the higher stimulation frequencies displayed significant force losses after the first fatiguing protocol.

From Fig. 3, it is evident that as fatigue gets greater, the force–frequency relationship becomes more and more flat; any increase in stimulation frequency increases the force output to a smaller extent than in less fatigued states. In the pre-fatigued state, there is ~ 409% increase in active force from 20 to 100 Hz, compared to only ~ 162%, ~ 140%, and ~ 123% increase for the first, second, and third levels of fatigue, respectively.

When comparing the percentage reduction from the pre-fatigued active force, there was, expectedly, a significant interaction between the level of fatigue and the frequency of stimulation (*p* < 0.001). Again, one-way repeated measures ANOVA tests to determine the simple main effects of fatigue level revealed a frequency dependence of fatigue. Table 1 illustrates that the percentage reduction in force from the prefatigued state increases as the stimulation frequency increases, for all levels of fatigue. At the first level of fatigue, there is a significant 24% *increase* in active force for the twitch contractions, as well as a non-significant increase in active force at 20 Hz, compared to a 47% reduction at 100 Hz. Similarly, at the second and third levels of fatigue, the percentage reduction for 20 Hz was 26% and 59%, respectively, compared to 65% and 82% reduction at 100 Hz. It seems clear that lower frequency contractions seem to be less affected by fatigue than contractions at higher frequencies. To get a gauge of prolonged fatigue, one can look at the percentage reduction in force between the pre-fatigued state and after 30 min of recovery. Interestingly, while all frequencies exhibited some degree of force loss after the 30 min recovery period, the differences were *not* significant for the lower frequencies (1 Hz, 20 Hz, and 40 Hz), while the differences *were* significant for the higher frequencies (60 Hz, 80 Hz, and 100 Hz). While these statistical results may stem from a relatively small sample size and increased variance with the lower frequency contractions, it does suggest that fatigue was not preferential to the low-frequency contractions.

**Table 1:** The percentage reduction in active force from the pre-fatigued active force at a given frequency.

Frequency (Hz)	Fatigue 1 (%)	Fatigue 2 (%)	Fatigue 3 (%)	Recovery (%)
1 Hz	− 24.10 ± 4.85*	10.32 ± 5.63	53.22 ± 5.79*	9.37 ± 3.89
20 Hz	− 2.46 ± 2.41	26.16 ± 4.27*	58.79 ± 4.20*	12.33 ± 5.12
40 Hz	23.35 ± 2.68*	43.83 ± 4.00*	64.34 ± 4.48*	7.22 ± 6.06
60 Hz	41.31 ± 2.02*	60.12 ± 2.70*	77.04 ± 2.75*	12.49 ± 2.09*
80 Hz	46.20 ± 0.89*	64.52 ± 1.49*	80.60 ± 1.93*	8.44 ± 1.60*
100 Hz	47.06 ± 1.27*	65.20 ± 1.79*	82.03 ± 2.75*	8.46 ± 1.72*

Values are mean ± SEM. Fatigue 1 = 1 contraction every 4 s. Fatigue 2 = 1 contraction every 2 s. Fatigue 3 = 1 contraction every second. Negative values refer to an increase in active force from the pre-fatigued condition.

*Denotes a significant change from the pre-fatigued value at a given frequency.

## Discussion

From our data, it is clear that during fatiguing contractions, there is a frequency dependence of force reduction, whereby contractions at lower frequencies are “protected” from fatigue compared to contractions with higher frequency stimulation. We saw, at a given level of fatigue, that as the frequency of stimulation increased, the relative reduction in force was greater. Notably, we even saw *increases* in force output at the lowest frequencies during the first level of fatigue, delaying the apparent reduction in force at these lower frequencies compared to the higher frequencies. Additionally, there were differences in the apparent degree of prolonged fatigue, whereby only the higher stimulation frequencies maintained a significant decrease in force after the 30-min recovery period.

**Figure 4:**
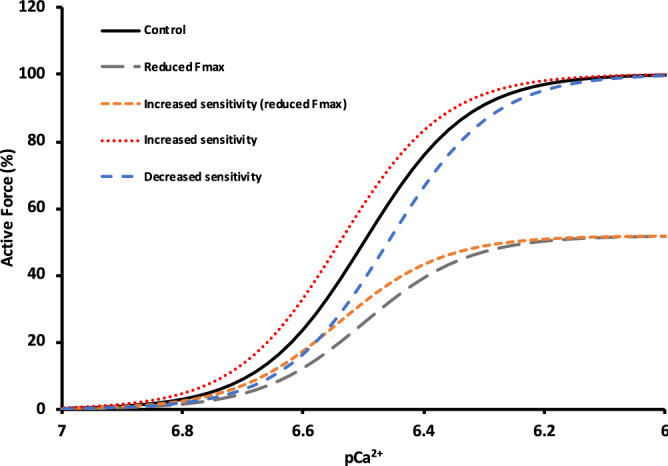
Hypothetical force–pCa relationship for a control muscle, a muscle with increased Ca^2+^ sensitivity, and a muscle with decreased Ca^2+^ sensitivity based on MacIntosh and Rassier^3^. Additionally, hypothetical force–pCa curves assuming that force per cross-bridge has decreased due to fatigue are shown (bottom two curves). Note that if force per cross-bridge is reduced due to fatigue such that peak force is ~ 50% of baseline, increased Ca^2+^ sensitivity would allow the resultant force outputs to be proportionately greater relative to baseline at the lower Ca^2+^ concentrations than at higher concentrations (compare orange square dotted curve to solid curve). This is similar to what we found in regard to lower frequencies having less relative force reduction than higher frequencies of stimulation.

In regards to what exactly is causing the fatigue that we observed, there are essentially three potential explanations: decreased [Ca^2+^], decreased Ca^2+^ sensitivity, and a reduced force per cross-bridge^3^. Both decreased Ca^2+^ release, as well as decreased Ca^2+^ sensitivity will affect low-frequency contractions more than high-frequency contractions. Since Ca^2+^ release is proportional to the frequency of stimulation, a lower frequency of stimulation will result in a lower [Ca^2+^]. Due to the steepness of the force–pCa curve at the lower frequencies, any reduction in Ca^2+^ release will result in a large drop in force. Because the curve becomes less steep at the higher ends, a reduction in Ca^2+^ release will result in a smaller drop-off in force. This can be visualized in Fig. 4. A decrease in Ca^2+^ sensitivity will similarly affect force output more so at lower frequencies of stimulation compared to higher frequencies. This is because at higher frequencies, any effects of increased or decreased Ca^2+^ sensitivity become smaller due to the flattening of the force–[Ca^2+^] relationship. In either case, these mechanisms of fatigue would preferentially affect lower frequencies of stimulation. Because our data showed that the lower frequencies of stimulation had the *smallest* reductions in force during fatiguing contractions, this means that either activity-dependent potentiation was disguising the effects of fatigue at these frequencies, or perhaps decreased Ca^2+^ release or decreased Ca^2+^ sensitivity were not the only factors in the fatigue. Considering recent work by Olsson et al^26^., there may be no decrease in Ca^2+^ sensitivity at 37 °C. The other potential mechanism in the reduction in force during the fatiguing contractions is a reduced force per cross-bridge. In this regard, Lou and Edman showed that there was a reduction in force per cross-bridge during fatiguing contractions of isolated frog muscle^27^. As well, Nocella et al. reported reductions in force per cross-bridge during a fatiguing protocol in mouse fibre bundles, and attributed this reduction to rises in P_i_^28^, which would be expected to increase as fatigue progressed^29^. While we did not measure stiffness in our experiments which makes any speculation on this mechanism difficult, considering these reports, as well as the data we presented, it is an interesting consideration.

**Figure 5:**
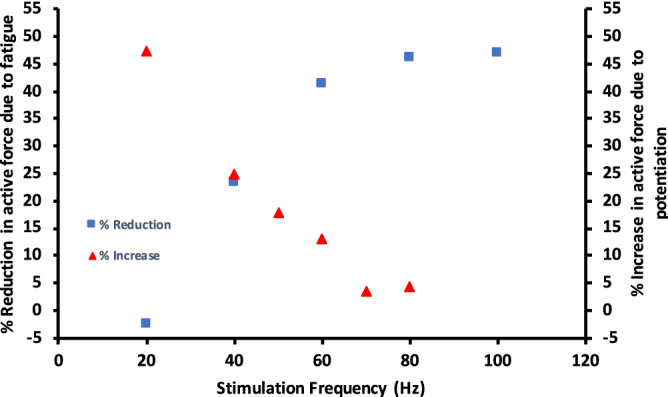
Data from MacIntosh and Willis^31^ combined with data from our experiments. The percentage reduction in active force was from the first fatiguing protocol in our experiments, and the percentage increase was from the data of MacIntosh and Willis. Note the reciprocal relationship between the percentage increase in active force due to potentiation, and the percentage reduction in active force due to fatigue at each frequency.

The fact that we observed increases of force at the lower frequencies during early fatigue points towards activity-dependent potentiation being a factor in our results. The interpretation of the finding that the relative reduction in force was greater at higher stimulation frequencies is that activity-dependent potentiation is relatively greater at lower frequencies, allowing the reduction in force by fatigue to be compensated for by potentiation in a frequency-dependent manner. This explanation seems reasonable, based on the findings of Fowles and Green^23^, and Rassier and MacIntosh^30^, who showed that low-frequency fatigue can coexist with potentiation. Additionally, MacIntosh and Willis^31^ showed that, when submaximal quintuplet contractions were repeated twice per second for 7 s, the relative increase in force between 0 and 7 s was greater with lower stimulation frequencies (see Fig. 5). These data demonstrate the frequency dependence of potentiation, whereby relative force increases are greater at the lower frequencies and gradually decrease as the stimulation frequency is increased.

The mechanism of activity-dependent potentiation is RLC phosphorylation, which is thought to increase Ca^2+^ sensitivity by increasing the mobility of the myosin head, positioning it further away from the myosin backbone, and closer to the actin molecule^20^. High levels of Ca^2+^ can also increase the mobility of the myosin head on its own^21^, and so at higher levels of activation, any RLC phosphorylation provides little “additional” myosin head mobility, and thus the relative enhancement of force at the higher frequencies is less apparent. Therefore, this increased Ca^2+^ sensitivity is expected to have a relatively greater effect at lower levels of activation^31^, consistent with the results shown here.

Figure 5 shows the comparison between the percentage increase in active force at each stimulation frequency from MacIntosh and Willis^31^, with the percentage decrease in active force during the first fatiguing protocol of our experiments. At lower frequencies (20 Hz and 40 Hz), the percentage increase in active force due to potentiation is high; concurrently, at these same frequencies, the percentage reduction in active force during fatiguing contractions is low. Alternatively, at the higher stimulation frequencies (60–100 Hz), the percentage increase in active force due to potentiation is low, and the percentage reduction due to fatigue is high. Once again, the interpretation of this data is that potentiation “protects” against fatigue in a frequency dependent manner; at lower stimulation frequencies, potentiation plays a relatively greater role in maintaining force production, whereas at higher stimulation frequencies, potentiation plays a negligible role and thus force output is reduced to a relatively greater extent. Considering that in vivo motor unit discharge rates are relatively low (< 50 Hz)^32,33^, this means that during fatigue, potentiation may play an important role in minimizing any force reduction that does occur. Thus, potentiation via RLC phosphorylation may be an important factor to maintain force production during fatiguing activities involving prolonged intermittent contractions such as cycling or running.

From our data, we can conclude that during fatiguing contractions, the relative degree of force loss is dependent upon the stimulation frequency, whereby higher frequency contractions exhibited proportionately greater losses of force than lower frequency contractions. While we cannot state the precise mechanism of fatigue, the fact that there was no clear evidence of low-frequency fatigue (which would suggest reduced Ca^2+^ release), adding to the fact that the higher frequency contractions were depressed to a larger extent, perhaps suggests a role for reduced force per cross bridge in the fatigue we observed. The results of these experiments suggest that there is an interplay of potentiation and fatigue that occurs during fatiguing contractions, and the relative effect of each of them is dependent on the frequency of stimulation. These experiments have confirmed the hypothesis that during fatiguing contractions, the relative reduction in force is dependent on the frequency of stimulation, and the likely mechanism for this is RLC phosphorylation, which increases force output in a frequency-dependent manner. Further research on the exact mechanisms behind the reduction in force output would lead to a more complete understanding of the current data.
